# Impact of PCSK9 Immunization on Glycemic Indices in Diabetic Rats

**DOI:** 10.1155/2021/4757170

**Published:** 2021-08-31

**Authors:** Amir Abbas Momtazi-Borojeni, Mahmoud Reza Jaafari, Elham Abdollahi, Maciej Banach, Amirhossein Sahebkar

**Affiliations:** ^1^Nanotechnology Research Center, Pharmaceutical Technology Institute, Mashhad University of Medical Sciences, Mashhad, Iran; ^2^Department of Medical Biotechnology, Faculty of Medicine, Mashhad University of Medical Sciences, Mashhad, Iran; ^3^Iran's National Elites Foundation, Tehran, Iran; ^4^Biotechnology Research Center, Pharmaceutical Technology Institute, Mashhad University of Medical Sciences, Mashhad, Iran; ^5^Department of Gynecology, Woman Health Research Center, Mashhad University of Medical Sciences, Mashhad, Iran; ^6^Department of Hypertension, Medical University of Lodz (MUL), Poland; ^7^Cardiovascular Research Centre, University of Zielona-Gora, Zielona-Gora, Poland; ^8^Applied Biomedical Research Center, Mashhad University of Medical Sciences, Mashhad, Iran; ^9^School of Pharmacy, Mashhad University of Medical Sciences, Mashhad, Iran

## Abstract

**Methods:**

To prepare the anti-PCSK9 vaccine, a peptide construct called Immunogenic Fused PCSK9-Tetanus (IFPT) was linked to the surface of nanoliposome carriers. Healthy rats received four subcutaneous injections of the vaccine at biweekly intervals. Two weeks after the last vaccination, anti-PCSK9 antibody titers, PCSK9 targeting, and inhibition of PCSK9–low-density lipoprotein receptor (LDLR) interaction were evaluated. After verification of antibody generation, the immunized rats were intraperitoneally treated with a single dose (45 mg/kg) of streptozotocin (STZ) to induce diabetes mellitus. The levels of fasting blood glucose (FBG) were measured, and the oral glucose tolerance test (OGTT) as well as the insulin tolerance test (ITT) were carried out to assess glycemic status. At the end of the study, the total cholesterol, low-density lipoprotein cholesterol (LDL-C), triglyceride, and high-density lipoprotein cholesterol concentrations were assayed. Histopathology examination of the liver and pancreas was also performed using the hematoxylin-eosin staining method.

**Results:**

The prepared nanoliposomal vaccine could strongly induce anti-PCSK9 antibodies in the vaccinated rats. Within one week following the STZ injection, the FBG level was lower in the vaccinated group vs. diabetic control group (49% (−171.7 ± 35 mg/dL, *p* < 0.001)). In the OGTT, the injected rats showed improved glucose tolerance as reflected by the reduction of blood glucose levels over 180 min, compared with the diabetic controls. Moreover, the ITT demonstrated that, after the insulin injection, blood glucose concentration declined by 49.3% in the vaccinated group vs. diabetic control group. Expectedly, the vaccinated rats exhibited lower (-26.65%, *p* = 0.03) plasma LDL-C levels compared with the diabetic controls. Histopathology examination of pancreas tissue demonstrated that the pancreatic islets of the vaccinated rats had a slight decline in the population of *β*-cells and few *α*-cells. Normal liver histology was also observed in the vaccinated rats.

**Conclusion:**

PCSK9 inhibition through the liposomal IFPT vaccine can improve the glucose and insulin tolerance impairments as well as the lipid profile in diabetes.

## 1. Introduction

Proprotein convertase subtilisin/kexin type 9 (PCSK9) is a plasma protein that is majorly generated and released by the liver cells. The liver-secreted PCSK9 is principally known for its role in the regulation of low-density lipoprotein (LDL) receptor (LDLR) on the surface of hepatocytes and, thereby, hemostasis of LDL cholesterol (LDL-C) in the bloodstream [1]. The plasma circulating PCSK9 controls the hepatic LDLR *via* posttranslational modification through targeting the extracellular domain of LDLR, epidermal growth factor-like repeat A (EGF-A), and subjecting it to the lysosomal digestion [2]. Indeed, EGF-A is a responsible domain for the LDLR recycling to the cell surface [3–5], and PCSK9 binding impedes the regular comeback of the LDLR to the cellular membrane and facilitates its digestion in lysosome compartments, leading to reduced liver clearance of the plasma LDL-C [6, 7].

Notably, patients with diabetes mellitus (DM) suffer from atherogenic dyslipidemia identified by increased LDL-C, hypertriglyceridemia, and decreased antiatherogenic high-density lipoprotein (HDL) particles [8, 9]. LDL-C has been found as a therapeutic target in diabetic dyslipidemia, and LDL-reducing approaches have been shown to reduce cardiovascular (CV) events in individuals with diabetes [10]. Statins are the main LDL-lowering agents, which act through suppression of cholesterol biosynthesis [11] plus a multitude of pleiotropic effects [12–18]. Although statin therapy shows strong effectiveness in ameliorating the CV endpoint events [19–21], many meta-analyses of randomized controlled trials reveal a documented link of the statin use to the elevated chance of new-onset DM [22–26].

PCSK9 inhibitors, mainly monoclonal antibodies (mAbs), provide a strong LDL-reducing approach that, in combination with statins at maximally tolerated doses, can decrease LDL-C approximately by 73% [27, 28] and decrease CV outcomes [29–31]. Although data from various clinical trials firmly show an appreciable mitigating effect of the PCSK9 inhibitor evolocumab on hyperlipidemia in T2DM patients [32], there are still some concerns regarding the correlation between PCSK9 inhibitors and DM complications [33–36]. Therefore, it is essential to evaluate the impact of a newly developed PCSK9 inhibitor on glycemic indices and the progression of diabetes.

To address this, preclinical and clinical studies in diabetic models and individuals are inevitable. Our previous study showed that a nanoliposomal vaccine targeting PCSK9 could significantly induce the generation of antibodies inhibiting the plasma PCSK9 and thereby reducing the plasma LDL-C in an experimental model of atherosclerosis [37–40]. To understand the effect of anti-PCSK9 therapy on glycemic indices, we evaluated the preventive impact of the nanoliposomal anti-PCSK9 vaccine in rats with streptozotocin- (STZ-) induced diabetes.

## 2. Methods

### 2.1. Nanoliposomal Vaccine Preparation and Characterization

Nanoliposomal vaccine embracing an immunogenic peptide conjugated to the surface of liposome nanoparticles was constructed according to the previously described method [41]. In brief, the lipid film hydration method was employed to provide liposome nanoparticles. A peptide construct containing PCSK9 and tetanus epitopes termed immunogenic fused PCSK9-tetanus (IFPT) peptide was attached to the surface of the prepared nanoparticles using the postinsertion method. The linkage efficiency and peptide content of the prepared liposomal IFPT (L-IFPT) formulation were measured by HPLC (high-performance liquid chromatography) analysis (Knauer; Berlin, Germany). Physical properties of the peptide-linked liposomes, including particle size, charge, and homogeneity, were assessed by the dynamic light scattering (DLS) approach on a Zetasizer (Nano-ZS, Malvern, UK). The verified L-IFPT formulation was adsorbed to 0.4% alum adjuvant (Sigma-Aldrich) at the 1 : 1 (*v* : *v*) ratio and used for in vivo study on STZ-induced diabetic rats.

### 2.2. The Animal

A total of 24 male Wistar Albino rats (179 ± 5.5 g) were provided by the Laboratory Animal Research Center of the Faculty of Medicine Mashhad University of Medical Sciences, Mashhad, Iran. All animal handling procedures were conducted strictly based on the animal welfare guidelines approved by the Institutional Ethics Committee and Research Advisory Committee. Rats were weighed weekly and at the end of the experiment, located in an air-conditioned space at a room temperature of 22 ± 2°C with 12 : 12 h light/dark cycle, and fed a standard rodent diet and water *ad libitum.* Upon starting the experiment and prior to STZ-induced diabetes, rats were administered either with vaccine formulation or saline buffer. The tail vein blood collection was carried out two weeks following the last immunization for the titration of the plasma anti-PCSK9 antibody, and after STZ injection for the FBG measurement. At the end of the experiment, rat euthanasia was performed by intravenous injection (30 mg/kg) of thiopental sodium [42, 43], and blood was collected *via* heart puncture to check out the plasma lipid profile. Pancreas and liver tissues were isolated to determine their weight and cell destruction.

### 2.3. The Vaccination Scheme

**Figure 1:**
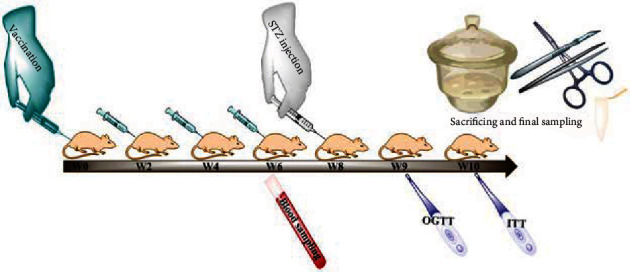
Schematic of animal interventions during the study.

One week before the study, the rats were domesticated to be randomly subjected into two groups, a vaccine-treated group (*n* = 8, 208 ± 6.14 g) and a nontreated group (*n* = 16, 207 ± 15.4 g). The vaccine group rats were biweekly immunized 4 times subcutaneously (s.c.) with a 200 *μ*L L-IFPTA formulation containing 20 *μ*g peptide, while nontreated group rats simultaneously received saline buffer. The time point of the first immunization is referred to as week 0 (W0). Three boosters were then implicated at W2, W4, and W6 (Figure 1). The blood was withdrawn at the time point W6 (Figure 1), and the plasma samples were prepared and used for antibody titer analysis. The vaccination schedule, including the dose and the duration, was planned based on our previous study [41].

### 2.4. Evaluating the Efficacy of Nanoliposomal Anti-PCSK9 Vaccine in Rats

To determine the efficacy of the liposomal vaccine in rats, the plasma anti-PCSK9 antibody titer, the plasma PCSK9 concentration, antibody-targeted PCSK9, and antibody-inhibited PCSK9/LDLR interaction were analyzed as explained in our recent study [41].

Concisely, the liposomal vaccine-induced anti-PCSK9 antibody was evaluated by the ELISA method using serially diluted plasma (1 : 4 × 1 : 400). A microwell plate reader (Sunrise, Tecan, Switzerland) was employed to detect the optical density (OD) at 450 nm. The dilution factor attributed to 50% of the maximal optical density (OD_max_/2) was measured to define the antibody titer [41]. To quantify the level of the free plasma PCSK9 in vaccinated mice, a PCSK9 ELISA kit (CircuLex™, Cy-8078, MBL, Woburn, MA) was employed as instructed by the manufacturer. This PCSK9 ELISA kit was also used to assay the interaction of vaccine-produced antibodies with PCSK9 and, thereby, determine inhibition of the rat plasma PCSK9 by the generated antibodies [41]. To find the functionality of the induced antibodies, the ability of the vaccinated rat's plasma for inhibition of the PCSK9-LDLR interaction *in vitro* was assessed by a PCSK9-LDLR *in vitro* binding assay kit (CircuLex™, Cy-8150, MBL, Woburn, MA). The higher ELISA OD shows a higher amount of PCSK9-LDLR interaction, in which in the presence of anti-PCSK9 antibodies, such interaction is impeded and, as a result, detected ELISA OD is reduced [41].

### 2.5. STZ-Induced T1DM

14 days following the last vaccination (W8, when the antibody titer was at the peak level, based on our previous finding [41]), both vaccine-treated and nontreated groups were subjected to a diabetes experiment to evaluate the antidiabetic effects of the anti-PCSK9 vaccine. Therefore, T1DM condition was induced in overnight-fasted (12 h) rats by a single intraperitoneal injection of STZ (45 mg/kg, Sigma-Aldrich) freshly dissolved in citrate-buffered saline (0.1 M, pH 4.5) [44]. The rats in the nonvaccinated group were randomized into two groups: the normal control (NC) group (*n* = 8; unvaccinated and received citrate buffer) and the diabetic control (DC) group (*n* = 8; unvaccinated and received STZ). The vaccine-treated group (*n* = 8) received STZ and was assigned as the vaccinated STZ-injected (VS) group. The first week after STZ injection, rats in the DC group had FBG concentrations > 250 mg/dL that confirmed the T1DM model [44].

### 2.6. Oral Glucose Tolerance Test (OGTT)

To assess the glucose tolerance ability of each rat, an OGTT was conducted on rats that were fasted overnight with a glucose dose of 2 g/kg at W9. Briefly, glucose solution was orally given, and blood glucose concentrations were checked by a glucometer (EasyGluco, South Korea) at time point 0 min (before glucose load) and 30, 60, 90, 120, 150, and 180 min after the oral glucose load [45]. The resultant data were expressed as an integrated area under the curve for glucose (AUC_glucose_), which was calculated by trapezoid rule using GraphPad Prism version 7.04.

### 2.7. Insulin Tolerance Test (ITT)

An insulin tolerance test was performed to determine the measure of peripheral utilization of glucose. At W10, insulin (0.8 U/kg) was intraperitoneally administered to overnight-fasted rats. Blood glucose was measured at time point 0 min (before the insulin injection) and 15, 30, 45, 60, 75, 90, and 120 min after the insulin injection [46]. The results were expressed as AUC_glucose_.

### 2.8. Lipid Profile Analysis

The plasma levels of LDL-C, HDL cholesterol (HDL-C), triglyceride (TG), and total cholesterol (TC) were assessed at the end of the study (W10) with commercial kits (BioSystems) as instructed by the manufacturer.

### 2.9. Histopathology Examination

At last, rats were euthanized, and organ samples were collected. Immediately upon removal, small pieces of the isolated pancreas and liver tissues were cut and immersion fixed in 10% buffered formalin. The formalin-embedded tissues were gradually dehydrated, embedded in paraffin, cut into 5 *μ*m sections, deparaffinized, and eventually stained using the hematoxylin and eosin (H&E) method. The histology of H&E-stained sections was checked out by an expert pathologist, using light microscopy supplied with a digital camera under a magnification of 400x.

### 2.10. Statistical Analysis

GraphPad Prism (version 7.04) and IBM SPSS Statistics for Windows, version 20 (IBM Corp., Armonk, NY, USA) were used for statistical analysis. The results were analyzed using the one-way ANOVA and Bonferroni post hoc multiple comparison test to evaluate the significance of the differences between the animal groups. Values were expressed as the mean ± SD or the mean ± SEM, lower-upper 95% confidence interval of the mean. Results with *p* < 0.05 were considered as statistically significant.

## 3. Results

### 3.1. Nanoliposomal Formulation

The empty nanoliposomes and the IFPT-linked nanoliposomes were found to have a size range from 150 nm to 180 nm in diameter, in which the polydispersity index was <0.2, revealing the preparation of nanovesicles with high homogeneity. Analysis of surface charge also showed that the prepared formulations had negative zeta potential. As revealed by HPLC analysis, 96% of IFPT peptides added at the beginning were linked to liposome nanoparticles.

### 3.2. Efficacy of Liposomal Anti-PCSK9 Vaccine in Rats

**Figure 2:**
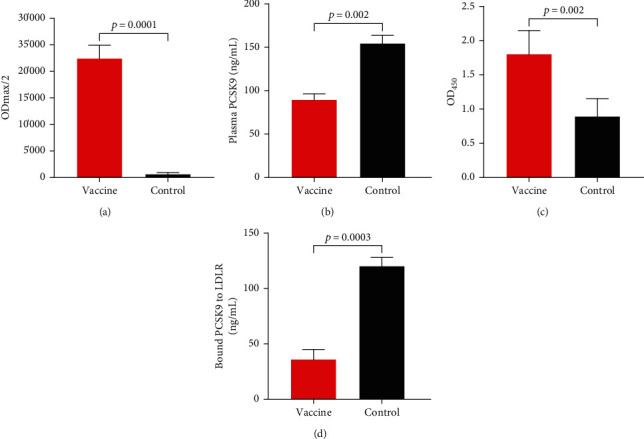
The function of liposomal anti-PCSK9 vaccine in rats upon 4 vaccinations. (a) Anti-PCSK9 peptide IgG titers (OD_max_/2) in vaccinated and control rats (*n* = 8/group). (b) Concentrations of the plasma PCSK9 in vaccinated and control rats were 89 ± 7 ng/mL and 154 ± 10 ng/mL, respectively (*n* = 3 replicates of the pooled samples of 8 rats per group). (c) Direct detection of vaccine-generated anti-PCSK9 antibodies targeting plasma PCSK9. Increased OD_450_ is indicative for evaluating the direct binding of anti-PCSK9 antibodies to plasma PCSK9 from vaccinated and control rats (*n* = 3 replicates of the pooled samples of 8 rats per group). (d) *In vitro* evaluation of PCSK9/LDLR interaction. Plasma sample of vaccine rats contained vaccine-generated anti-PCSK9 antibodies that could decrease PCSK9 interaction to LDLR by 30% when compared with the plasma sample of control rats (*n* = 3 replicates of the pooled samples of 8 rats per group). Bars show mean values, error bars show ±SD.

The L-IFPTA vaccine could induce a high-titer IgG antibody against the PCSK9 peptide in rats upon 4 vaccinations in biweekly intervals (Figure 2(a)). Vaccine-generated anti-PCSK9 antibodies showed specific targeting of the plasma PCSK9 in the vaccinated rats. As demonstrated in Figure 2(b), the plasma concentrations of the free PCSK9 in the vaccine group (89 ± 7 ng/mL) were significantly (*p* = 0.002) lower than that in the control group (154 ± 10 ng/mL). The plasma PCSK9 concentration was significantly reduced by 57.8% in the vaccinated rats when compared with the control rats. As revealed by the PCSK9 inhibition assay using the ELISA method, the plasma of vaccinated rats could emerge as a noticeably higher OD_450_ signal than that of the control rats (Figure 2(c)), showing a direct and specific targeting of the plasma PCSK9 by liposomal vaccine-induced anti-PCSK9 antibody in rats. Additionally, the induced antibodies could markedly inhibit *in vitro* binding of PCSK9 to LDLR, showing the functionality of the induced antibodies. Of note, it was found that in the attendance of the vaccinated rat's plasma, *in vitro* interaction of murine PCSK9 and LDLR was significantly hindered by 30%, compared with the plasma sample of the control group (Figure 2(d)). In sum, the L-IFPTA vaccine could induce specific and functional antibodies that inhibit PCSK9-LDLR interaction through specific targeting of the plasma PCSK9 in rats.

### 3.3. Body Weight Change

**Figure 3:**
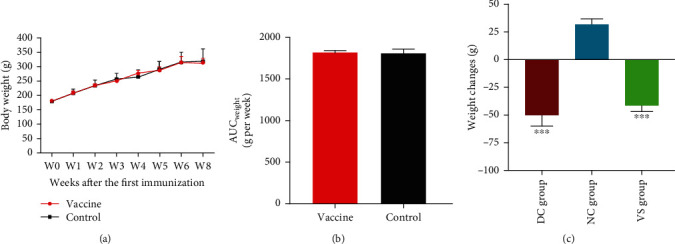
The body weight gain (a) and corresponding areas under the weight curve (AUC _weight_) (b) in the vaccine-treated and nontreated control groups during the vaccination period, from week 0 (W0) to week 8 (W8). Data are expressed as the mean ± SD. (c) The body weight changes two weeks after STZ injection in the vaccinated STZ-injected (VS), diabetic control (DC), and the normal control (NC) rats, from week 8 to week 10. Data are expressed as the mean ± SEM. ^∗∗∗^*p* < 0.001 compared to the NC group.

The body weight changes during the vaccination period in the vaccine-treated (V) and nontreated control (C) groups were calculated by initial weight (W0) subtraction from the final weight of the animal at the time point of STZ injection (W8). The body weight was significantly raised in the V group (129.5 ± 8.87 g weight gain, *p* < 0.001) and the C group (138.5 ± 12.75 g weight gain, *p* < 0.001) (Figure 3(a)). Likewise, the integrated areas under the weight curve (AUC_weight_) over the vaccination period were not statistically different (*p* > 0.05) in the V group (1812 ± 22.75 g) and the C group (1806 ± 48 g) (Figure 3(b)). After STZ injection, body weight gain significantly failed in the VS and DC groups but not in the NC group. Hence, during the two weeks after STZ injection, the body weights of the VS and DC groups had significantly dropped by -16.17 ± 6.3% (−50.56 ± 21 g, *p* = 0.009) and −14.35 ± 4% (−41.35 ± 10 g, *p* = 0.01), whereas the NC group showed a significant body weight gain by 10 ± 2.4% (31.85 ± 9.8 g, *p* = 0.02) (Figure 3(c)).

### 3.4. Liposomal Anti-PCSK9 Vaccine Reduces FBG Levels

**Figure 4:**
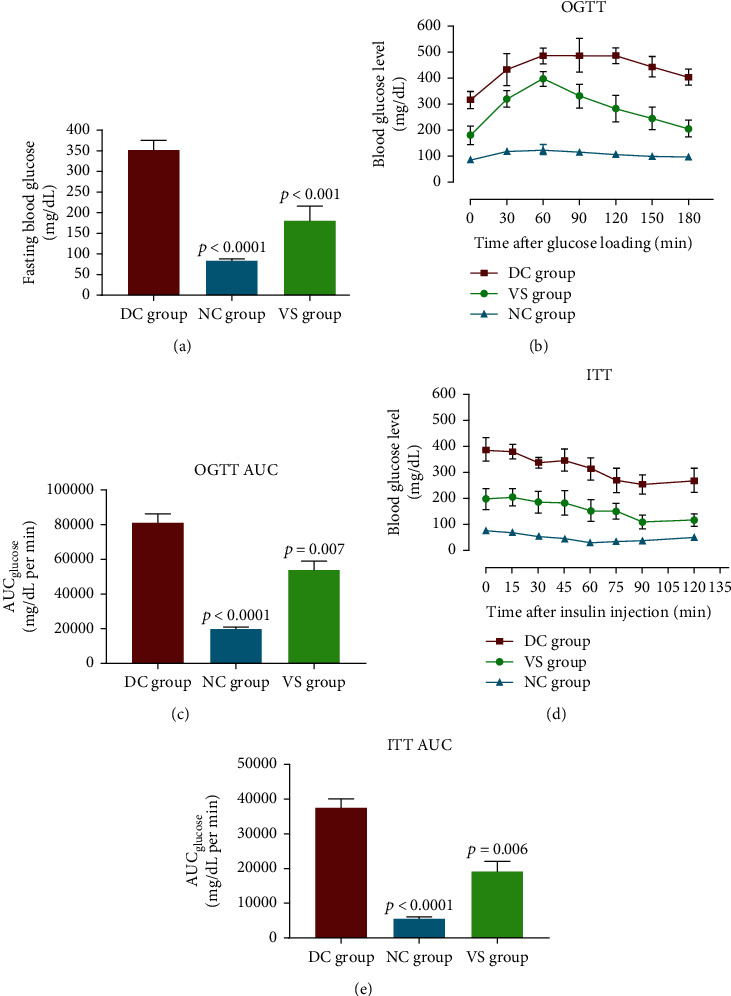
The levels of fasting blood glucose (FBG) in the vaccinated STZ-injected (VS), diabetic control (DC), and normal control (NC) rats during the week after STZ injection. Data are represented as the mean ± SEM. ∗∗∗ indicates *p* < 0.0001 compared to both the VS and NC groups (a). Oral glucose tolerance test (OGTT) (b) and corresponding areas under the glucose curve (AUC_glucose_) over 180 min (c) following feeding (2 g/kg) of the vaccinated STZ-injected (VS), diabetic control (DC), and normal control (NC) rats with oral glucose. Data are represented as the mean ± SD. The mean values of AUC_glucose_ in the VS and DC groups showed a significant difference (*p* < 0.0001) compared to the NC group. When compared to the DC group, the mean values of AUC_glucose_ in the VS group were significantly (*p* = 0.007) different. The insulin tolerance test (ITT) (d) and corresponding areas under the glucose curve (AUC_glucose_) over 120 min (e) following insulin administration (0.8 U/kg) in the vaccinated STZ-injected (VS), diabetic control (DC), and normal control (NC) rats. Data are represented as the mean ± SD. The mean values of AUC_glucose_ in the VS and DC groups showed a significant difference (*p* < 0.0001) compared to the NC group. When compared to the DC group, the mean values of blood glucose levels and AUC_glucose_ in the VS group were significantly (*p* = 0.006) lower.

Within one week following STZ injection, the FBG measurement indicated that the DC rats suffered a significant (*p* < 0.0001) hyperglycemia (351.7 ± 23.58 mg/dL, 95% CI: 301-402 mg/dL) compared to the NC group (87 ± 2 mg/dL, 95% CI: 82-91 mg/dL), verifying STZ-induced diabetes mellitus. Interestingly, there was no significant increase in the FBG levels in the VS group (180 ± 36 mg/dL, 95% CI: 94-266 mg/dL) in comparison to the NC group. The FBG level was 49% (−171.7 ± 35 mg/dL, *p* < 0.001) lower in the VS group versus the DC group (Figure 4(a) and Table 1).

### 3.5. Liposomal Anti-PCSK9 Vaccine Improves Glucose Sensitivity

To evaluate glucose sensitivity in the vaccinated STZ-injected rats (VS), OGTT was performed one week after STZ injection (W8). Oral glucose administration (2 g/kg) in the DC rats showed a significant elevation in the blood glucose levels (after 60 min) and exhibited that exogenous glucose administration significantly impaired glucose tolerance compared to the NC rats. The VS rats had significantly improved glucose tolerance ability compared to the DC rats. Further, the VS rats recorded a significant reduction in the level of blood glucose over a period of 180 min compared to the DC rats (Figure 4(b)). The integrated area under the glucose curve (AUC_glucose_) over 180 min of the DC rats was significantly (*p* < 0.0001) higher than that of the NC rats. The measurement of AUC values demonstrated that blood glucose levels were significantly (*p* = 0.007) decreased by 34.5% in the VS rats compared to the DC rats (Figure 4(c)). In the VS rats, glucose levels after 60 min started a markedly decreasing trend to reach baseline levels at time 180, while in the DC group, a consistent level of glucose was indicated between 60 and 120 min. Although glucose levels had slowly dropped after 120 min in the DC rats, it did not reach baseline levels at 180 min (Figure 4(b)).

### 3.6. Liposomal Anti-PCSK9 Vaccine Improves Insulin Sensitivity

To measure the insulin sensitivity, an insulin challenge (0.8 U/kg, i.p.) was performed four days after the OGTT. Blood glucose concentration and AUC_glucose_ in the DC group were significantly (*p* < 0.0001) higher at different time points after insulin administration compared to the NC rats. Blood glucose concentration and AUC_glucose_ in the VS rats were significantly (*p* = 0.006) lower during ITT in comparison to the DC rats. Blood glucose concentrations in the VS rats were not significantly higher at 90 and 120 min after the insulin injection compared with the glucose levels at the corresponding time points in the NC rats (Figure 4(d)). As found by comparison of AUC values, blood glucose levels showed a 49.3% decrease in the VS group compared to the DC group (Figure 4(e)).

### 3.7. Liposomal Anti-PCSK9 Vaccine Reduces the Plasma LDL-C

**Figure 5:**
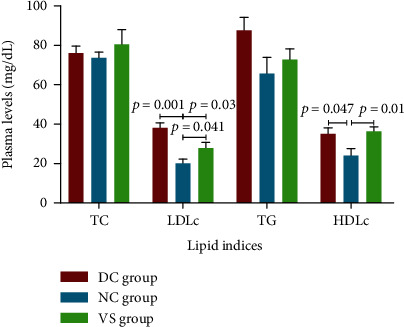
The plasma levels (mg/dL) of total cholesterol (TC), low-density lipoprotein cholesterol (LDL-C), triglyceride (TG), and high-density lipoprotein cholesterol (HDL-C) in the vaccinated STZ-injected rats (the VS group), diabetic control rats (the DC group), and normal control rats (the NC group) at the end of the study. *N* = 8. Data are expressed as the mean ± SEM.

At the end of the experiment, the measurement of lipid indices displayed no significant difference in the plasma TC and TG between different groups. No difference was indicated in the plasma HDL-C between the DC and VS groups. A significantly and appreciably raised LDL-C (90%, *p* = 0.001) and HDL-C (45.8%, *p* = 0.047) were found in the DC rats compared to the NC rats. The VS rats showed appreciably lower (-26.65%, *p* = 0.03) plasma LDL-C levels than those in the DC rats. Compared to the NC group, the VS rats exhibited significantly higher plasma levels of LDL-C (39.4%, *p* = 0.041) and HDL (51.56%, *p* = 0.01) (Figure 5).

### 3.8. The Relative Weights of the Pancreas and the Liver

The relative organ weights were measured as (organ weight/body weight) × 100, at the last week of the experiment (W10). Relative pancreas weights were 0.424 ± 0.045, 0.28 ± 0.039, and 0.376 ± 0.017 g/100 g body weight in the VS, DC, and NC rats, respectively. The relative pancreas weight of the VS group was significantly higher (51 ± 14.6%, *p* = 0.001) than that of the DC group, while there was no significant difference when compared to the NC group. However, the relative pancreas weights of the DC group showed a 34 ± 23% (*p* = 0.02) decrease compared to the NC group. In the case of the liver, the relative weights were found to be 3.33 ± 0.42, 3.73 ± 0.25, and 4.01 ± 0.62 g/100 g body weight in the VS, DC, and NC rats, respectively. Statistical analysis demonstrated that the relative liver weights were not significantly different between all experimental groups.

### 3.9. Pancreas Histopathology

**Figure 6:**
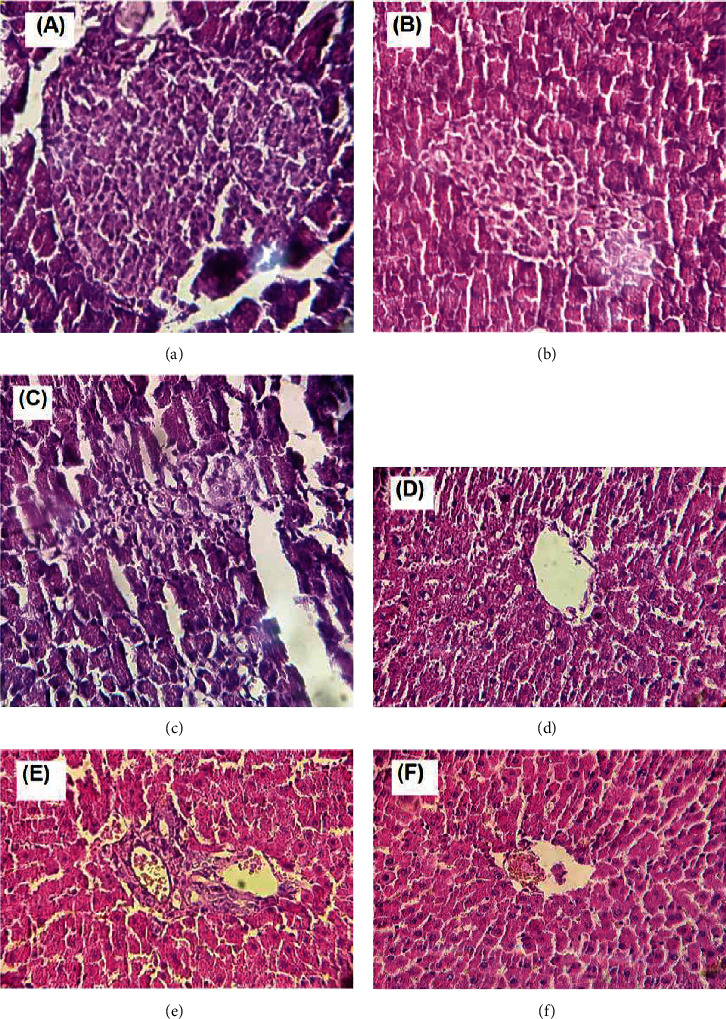
Histopathology of the pancreas in the normal control (NC) group (a), the diabetic control (DC) group (b), and the vaccinated STZ-injected (VS) group (c) at 400x magnification. Histopathology of the liver in the normal control (NC) group (d), the diabetic control (DC) group (e), and the vaccinated STZ-injected (VS) group (f) at 400x magnification.

The histopathology alterations in pancreas were exhibited after H&E staining in all rats (Figure 6). Microscopic examination of pancreas sections demonstrated the normal morphology and proportion of exocrine acinar architecture and Langerhans islets without evidence of cellular degeneration and necrosis, in the NC rats. Pancreatic islets stained lighter than the surrounding acinar cells. The normal islet cells showed predominantly insulin-producing *β*-cells with granular basophilic cytoplasm and few eosinophilic glucagon-producing *α*-cells. The acinar cells including pyramidal cells with apical acidophilic cytoplasm, which stained intensely, were settled in lobules with prominent basal nuclei (Figure 6(a)). In the DC rats, pathological alterations of both exocrine and endocrine compartments were observed. Swollen-acinar cells containing small vacuoles were noted. Interlobular ducts were lined by flattened epithelium. The pancreatic islets demonstrated a remarkably reduced population of basophilic *β*-cells and several eosinophilic *α*-cells. The islets contained eosinophilic shapeless deposits, suggestive of cellular necrosis. The endocrine pancreas demonstrated areas of degeneration and necrosis among the endocrine cells comprising the islets of Langerhans (Figure 6(b)). The pancreatic islet of the VS rats showed a slightly diminished population of *β*-cells and only a few *α*-cells. Again, cellular degeneration and necrosis were observed within the islets of Langerhans. Atrophic acinar cells were evident and the border between exocrine and endocrine compartments were notably less distinct. Overall, the VS pancreas exhibited a lesser intensity of eosin compared to the NC rats (Figure 6(c)).

### 3.10. Liver Histopathology

The H&E-stained slides of the liver in the NC, DC, and VS rats displayed the normal hepatic histological architecture consisting of hepatic lobules with a normal central vein. Each lobule was made up of radiating sheets, strands of polygonal hepatocytes forming a network around a central vein. Hepatocytes have well-defined cell borders with pink eosinophilic cytoplasm and mostly central single nuclei; inclusions were not found. There were no hemorrhagic areas or fibrosis evident (Figures 6(d)–6(f)).

## 4. Discussion

A direct association between DM and elevated risk of “atherosclerotic CV disease” has been documented [47, 48]. The most established therapeutic target for managing diabetes-related CV complications is LDL-C [10]. PCSK9 inhibition is a safe and effective LDL-lowering approach. However, experimental and Mendelian randomization investigations demonstrated that genetic variants of PCSK9 manifesting reduced LDL-C levels are accompanied with increased FBG levels and an elevated chance of DM [34–36]. Hence, the safety and efficacy of PCSK9 inhibitors, especially those apart from mAbs, regarding the regulation of glycemic indices in diabetes require further investigations.

Here, we demonstrated for the first time the impact of PCSK9 inhibition *via* the vaccination approach on STZ-induced DM in diabetic rats. Interestingly, the results showed that prophylactic administration of the anti-PCSK9 vaccine can drop LDL-C levels and protect against the progression of STZ-induced diabetes, which was related to a significant improvement of glycemic indices including FBG, OGTT, and ITT, together with lower histopathological changes in the liver and the pancreas tissues. The liposomal anti-PCSK9 vaccine was shown to decrease the plasma concentrations of functional free PCSK9 in diabetic rats through direct and specific targeting, which was associated with suppression of PCSK9/LDLR interaction, causing an alleviation of plasma LDL-C.

The LDL-lowering effect of the liposomal anti-PCSK9 vaccine has been also found in our other preclinical studies where the preventive [37, 38] and the therapeutic [39, 40] effects in hypercholesterolemic mice as well as the safety of the vaccine in healthy nonhuman primates [49] were evaluated. Such an effect has been similarly reported by the studies using other PCSK9 inhibiting vaccines, including the AFFITOPE®-based anti-PCSK9 vaccine [50, 51], a human recombinant protein-based anti-PCSK9 vaccine [52], and a vaccine comprised of virus-like particles displaying PCSK9 peptides [53]. Interestingly, the just-mentioned vaccine approaches significantly ameliorated hypercholesterolemia in mouse models, such as APOE∗3Leiden.CETP mice used by the AFFiRiS group for developing the AFFITOPE® vaccine [51].

STZ-induced diabetes is a model of DM characterized by hyperlipidemia and *β*-cell dysfunction, causing the insulin deficiency and subsequent hyperglycemia and loss of body weight in the experimental animals. In our study, markedly elevated blood glucose, as well as intemperate intake of food and water, were seen in STZ-treated rats (DC group), in comparison to the normal control rats (NC group). STZ-induced hyperglycemia was found to be inhibited in vaccinated rats (VS group). The FBG measurement revealed that the blood concentration of glucose in VS rats was not significantly different from the normal control, which was both markedly lower compared with DC rats. As shown by OGTT analysis, glucose tolerance was significantly impaired in the DC group compared to NC. The anti-PCSK9 vaccine protected VS rats against STZ-induced glucose intolerance and improved glucose sensitivity in the VS group when compared with the DC group. ITT assessment revealed that the insulin sensitivity in STZ-treated rats was profoundly decreased, and the PCSK9 vaccine inhibited such deficiency in VS rats, leading to the enhanced peripheral utilization of glucose *via* the anti-PCSK9 vaccination. Hence, STZ-treated rats on the insulin challenge did not show a significant drop in their blood glucose concentrations, underlying that these diabetic rats lost their peripheral insulin sensitivity and therefore could not use the exogenously administered insulin to decrease glucose concentrations. Such finding shows that the anti-PCSK9 vaccination can protect VS rats against STZ-induced insulin resistance.

Our findings can be supported by the Fourier trial [54] that showed the HbA_1c_ and fasting blood glucose levels are comparable among patients with diabetes, prediabetes, or normoglycaemia treated either with evolocumab or placebo. Moreover, results from a comprehensive analysis of several independent phase 3 clinical trials including subjects without DM show that alirocumab exerts no significant effect on the incidence of DM, or on fasting blood glucose (FBG) and HbA_1c_, in comparison to either ezetimibe or placebo after a 6–18-month follow-up period [55]. However, several Mendelian randomization studies have revealed that loss-of-function mutations in the *PCSK9* gene are correlated with lower LDL-C but higher plasma concentrations of fasting glucose and elevated risk of DM [34–36]. Besides, local deficiency—but not plasma levels—of PCSK9 has been shown to be responsible for overexpression of LDLR in pancreatic cells, which leads to increased intracellular cholesterol amount and *β*-cell damage [56]. These results suggest that anti-PCSK9 mAbs, which inhibit PCSK9 merely in the blood circulation, may exert no negative influence on the function of *β*-cells, while in the mentioned Mendelian study, the impact of global PCSK9 deficiency was evaluated.

Furthermore, weight loss is a hallmark of DM due to the destruction of structural proteins and muscle damage, which are complications of insulin deficiency. Lack of insulin-induced nutrient uptake promotes hyperphagia, while hyperglycemia induces polyuria and ensuing polydipsia. Although the anti-PCSK9 vaccine could protect the vaccinated rats against STZ-induced hyperglycemia and improved glucose hemostasis to the same extent as the control rats, the loss of body weight gain in the VS group was evident in a similar fashion to the DC group. Such contradictory effects of the vaccination on STZ-induced diabetes can be explained by the exacerbated lipolysis and the elevated lipid peroxidation leading to weight loss in STZ-treated rats [57].

In summary, the aforementioned findings suggest that LDL lowering *via* liposomal vaccine-induced anti-PCSK9 antibodies not only does not exert side effects on glycemic control but also can improve glycemic indices and insulin sensitivity in diabetic animals. The present findings call for additional studies in other experimental models of diabetes to confirm the positive impact of PCSK9 immunization on glycemic indices.

## Figures and Tables

**Table 1:** Statistical analysis^∗^ of FBG values in the different experimental groups, one week after STZ injection.

Groups	Mean 1 (mg/dL)	Mean 2 (mg/dL)	Mean difference	SE of difference	95% CI of difference	*p* value
DC vs. NC	351.7	87	264.8	32.6	184.5 to 345	<0.0001
VS vs. NC	180	87	93	37.8	-186.4 to 0.2	0.0506
VS vs. DC	180	351.7	-171.7	35	85 to 258	<0.0001

^∗^Statistical analysis was performed using one-way ANOVA and Tukey-Kramer's*post hoc* multiple comparison test. CI: confidence interval; DC: diabetic control group; FBG: fasting blood glucose; NC: normal control group; SE: standard error; VS: vaccinated STZ-injected group; vs.: versus.

## Data Availability

Data are available from the corresponding author on reasonable request.
